# Aloe‐emodin‐mediated antimicrobial photodynamic therapy against dermatophytosis caused by *Trichophyton rubrum*

**DOI:** 10.1111/1751-7915.13875

**Published:** 2021-06-24

**Authors:** Wenpeng Ma, Miaomiao Zhang, Zixin Cui, Xiaopeng Wang, Xinwu Niu, Yanyan Zhu, Zhihong Yao, Feng Ye, Songmei Geng, Chengcheng Liu

**Affiliations:** ^1^ Department of Pathogenic Microbiology & Immunology School of Basic Medical Sciences Xi'an Jiao Tong University Health Science Center 76 West Yanta Road Xi'an 710061 China; ^2^ Clinical Laboratory The Second Hospital of Weinan 2 East Chaoyang Street Weinan 714000 China; ^3^ Department of Infection The First Affiliated Hospital of College of Medicine Xi'an Jiao Tong University 227 West Yanta Road Xi'an 710061 China; ^4^ Department of Dermatology The Second Affiliated Hospital of College of Medicine Xi'an Jiao Tong University 157 Xi Wu Road Xi'an 710004 China; ^5^ Department of Clinical Medicine Hanzhong Vocational and Technical College 81 Zongying Town Hanzhong 723002 China

## Abstract

*Trichophyton rubrum* is responsible for the majority of dermatophytosis. Current systemic and topical antifungals against dermatophytosis are often tedious and sometimes unsatisfactory. Antimicrobial photodynamic therapy (aPDT) is a non‐invasive alternative suitable for the treatment of superficial fungal infections. This work investigated the photodynamic inactivation efficacy and effects of aloe‐emodin (AE), a natural photosensitizer (PS) against *T. rubrum* microconidia *in vitro*, and evaluated the treatment effects of AE‐mediated aPDT for *T. rubrum*‐caused tinea corporis *in vivo* and tinea unguium *ex vivo*. The photodynamic antimicrobial efficacy of AE on *T. rubrum* microconidia was evaluated by MTT assay. The inhibition effect of AE‐mediated aPDT on growth of *T. rubrum* was studied. Intracellular location of AE, damage induced by AE‐mediated aPDT on cellular structure and surface of microconidia and generation of intracellular ROS were investigated by microscopy and flow cytometry. The therapeutic effects of AE‐mediated aPDT against dermatophytosis were assessed in *T. rubrum*‐caused tinea corporis guinea pig model and tinea unguium *ex vivo* model. AE‐mediated aPDT effectively inactivated *T. rubrum* microconidia in a light energy dose‐dependent manner and exhibited strong inhibitory effect on growth of *T. rubrum*. Microscope images indicated that AE is mainly targeted to the organelles and caused damage to the cytoplasm of microconidia after irradiation through generation of abundant intracellular ROS. AE‐mediated aPDT demonstrated effective therapeutic effects for *T. rubrum*‐caused tinea corporis on guinea pig model and tinea unguium in *ex vivo* model. The results obtained suggest that AE is a potential PS for the photodynamic treatment of dermatophytosis caused by *T. rubrum*, but its permeability in skin and nails needs to be improved.

## Graphical abstract

The skin lesion of tinea corporis was greatly improved and *Trichophyton rubrum* cells on the infected skin surface were all killed after AE‐mediated aPDT treatment on guinea pig model. AE‐mediated aPDT had an effective therapeutic effect for tinea unguium in ex vivo model.

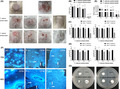

## Introduction

*Trichophyton rubrum* is a common dermatophyte in clinics which is responsible for the majority of dermatophytosis. It invades the human keratinous tissues such as hair, skin and nails to cause a range of superficial diseases such as tinea capitis, tinea corporis, tinea manus, tinea pedis, tinea cruris and tinea unguium (onychomycosis) (Li *et al*., 2021). Although these fungal infections are rarely life‐threatening, they are chronic, relapsing, and they can easily transmit from one patient to the another and significantly affect the patients’ quality of life (Fekrazad *et al*., 2017).

The current therapeutic options against dermatophytosis are dependent on the oral administration and topical application of antifungal drugs (Li *et al*., 2021). Although systemic antifungals are effective, they are still associated with adverse effects such as long duration of treatment, risk of hepatotoxicity, poor compliance of patients and possible serious drug interactions (Alberdi and Gómez, 2019). Moreover, long‐term and single use of these antifungals can induce the development of drug resistance (Baltazar *et al*., 2015). In contrast, topical agents are more desirable due to the low risk of side effects and drug interactions. However, these topical agents are still limited by their low treatment efficacy, long treatment session and high recurrence rate (Lipner and Scher, 2019). Thus, the development of novel topical technologies or therapeutic options against *T. rubrum*‐caused dermatophytosis is highly desirable.

As a promising and non‐invasive approach against bacteria, fungi, viruses and parasites, antimicrobial photodynamic therapy (aPDT) has attracted much attention for its unique reaction mode (St Denis *et al*., 2011). aPDT can effectively inactivate microbial cells in the presence of three essential elements: light with specific wavelength, photosensitizer (PS) and molecular oxygen through a phototoxic reaction. Light illumination stimulates PS from ground state to excited state that reacts with oxygen inside or around microbial cells and generates reactive oxygen species (ROS) (Dai *et al*., 2012), which can induce irreversible oxidative damage to their cellular structures and biomacromolecules, subsequently resulting in death (Calzavara‐Pinton *et al*., 2012). This unique inactivation mechanism makes aPDT preferable for the treatment of superficial infections, *for example* dermatophytosis.

With respect to conventional therapeutic regimes against dermatophytosis, aPDT exhibits high selectivity because phototoxic effects happen only in the area where light is administrated and PS accumulates in proliferative cells (such as dermatophytes) in a selective manner. aPDT shows high safety because fungi can be killed at combinations of PS and light doses much lower than that needed for a similar effect on keratinocytes, and all investigated PSs lack genotoxic and mutagenic activities (Calzavara‐Pinton *et al*., 2012). More importantly, due to the non‐specific oxidative mode of liberated ROS and oxidative damage on many intracellular targets (such as lipids, proteins and nucleic acids), it is generally believed that microbial cells cannot develop any mechanism of resistance (Calzavara‐Pinton *et al*., 2012; Kashef and Hamblin, 2017; Houang *et al*., 2018).

Several clinicians employed this novel technology to treat tinea pedis (Sotiriou *et al*., 2009a), tinea cruris (Sotiriou *et al*., 2009b) and tinea unguium (Piraccini *et al*., 2008) caused by *T. rubrum*. Results showed that aPDT was effective in the treatment of these dermatophytosis, and significant cure rates were obtained for patients who failed to standard medications or have contraindications to oral drugs. Additionally, aPDT was well tolerated by patients with only erythema, burning and mild pain (Simmons *et al*., 2015). In the reported clinical trials, the most frequently used PSs were 5‐aminolevulinic acid (5‐ALA), methyl aminolevulinate (MAL), methylene blue (MB) and rose bengal (RB).

**Fig. 1:**
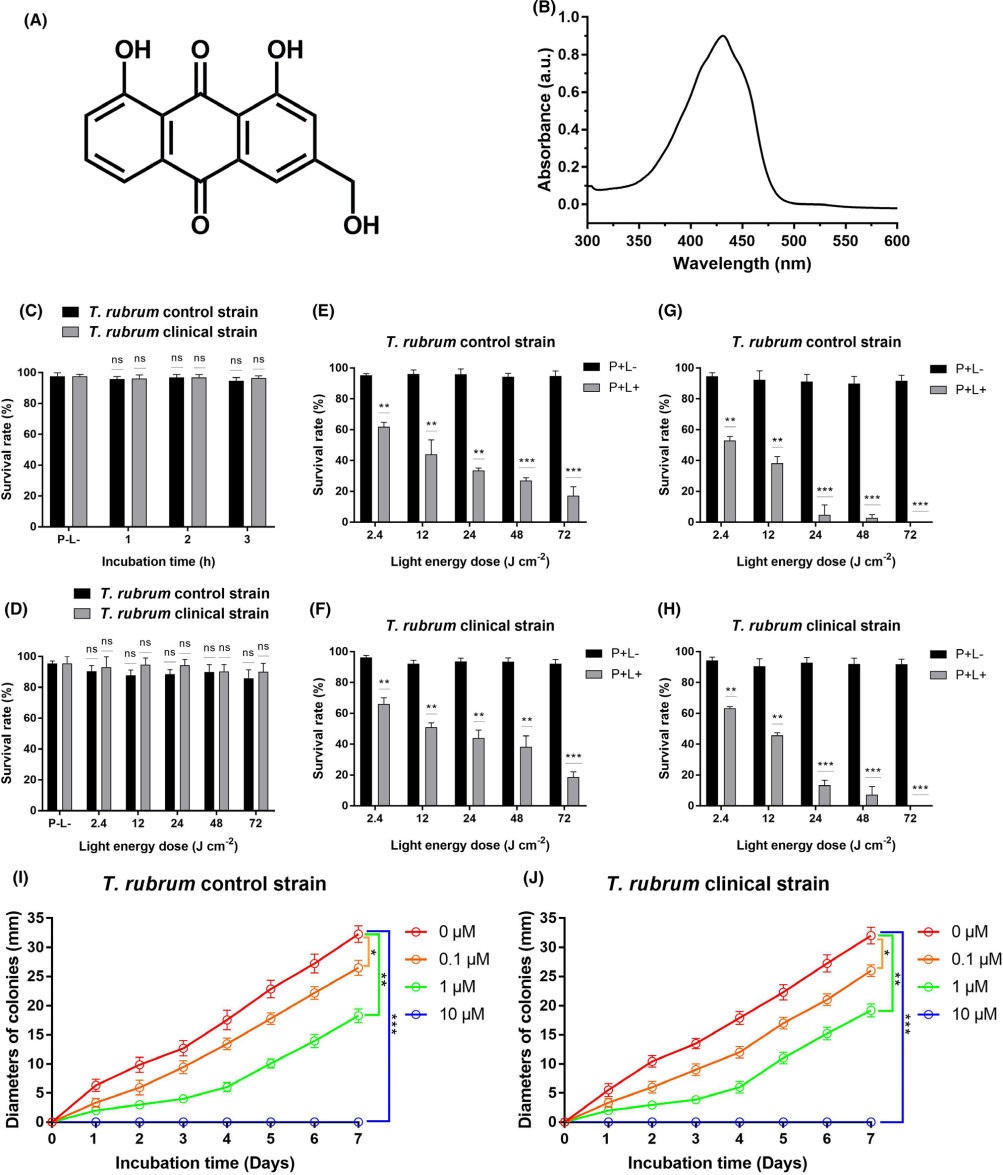
Photodynamic antimicrobial efficacy of AE on microconidia of *T. rubrum* control and clinical strains. A. Chemical structure of AE. B. Absorption spectra of AE. C. Survival rate of *T. rubrum* microconidia incubated for different durations with AE (10 μM) in the dark; (P−L−, P = photosensitizer, L = light irradiation): *T. rubrum* microconidia without any treatment. D. Survival rate of *T. rubrum* microconidia irradiated with different energy doses of 435 ± 10 nm light; (P−L−): *T. rubrum* microconidia without any treatment. E. Microconidial survival rate of control *T. rubrum* strain incubated with 1 μM of AE in the dark for 2 h and irradiated with different energy doses of light. F. Microconidial survival rate of clinical *T. rubrum* strain incubated with 1 μM of AE in the dark for 2 h and irradiated with different energy doses of light. G. Microconidial survival rate of control *T. rubrum* strain incubated with 10 μM of AE in the dark for 2 h and irradiated with different energy doses of light. H. Microconidial survival rate of clinical *T. rubrum* strain incubated with 10 μM of AE in the dark for 2 h and irradiated with different energy doses of light. I. Diameters of AE‐mediated aPDT‐treated control *T. rubrum* colonies after incubation at 28°C for 0–7 days (irradiated with 48 J cm^−2^ light). J. Diameters of AE‐mediated aPDT‐treated clinical *T. rubrum* colonies after incubation at 28°C for 0–7 days (irradiated with 48 J cm^−2^ light). Each value refers to mean ± standard deviation (SD) (*n* = 3) (ns: no significance, *P < 0.05, **P < 0.01, ***P < 0.001).

Besides these typical PSs, traditional Chinese herbs are rich resources of antifungals and promising PSs. Aloe‐emodin (AE, Fig. 1A) is a natural anthraquinone compound extracted from the widely used traditional Chinese herbs (such as *Aloe vera*, *Rheum palmatum*, *Cassia occidentalis* and *Polygonum multiflorum*). A large number of previous studies demonstrated that AE possessed many intrinsic therapeutic effects, including hepatoprotection, neuroprotection, anti‐cancer, anti‐inflammatory, anti‐bacterial, anti‐viral and anti‐parasitic activities (Dong *et al*., 2020). Recently, attentions have been focused on its photodynamic characteristics because the chemical structure of AE is quite similar to a well‐studied PS hypericin (Lin *et al*., 2017). Besides, the maximum absorption of AE is located in blue light region (Fig. 1B), which suggests that AE‐mediated aPDT is quite suitable for superficial infections (Zang *et al*., 2017). Therefore, this study evaluated the photodynamic inactivation efficacy and effects of this natural potential PS from traditional Chinese herbs against *T. rubrum* microconidia *in vitro* and investigated the treatment effects of AE‐mediated aPDT for *T. rubrum*‐caused tinea corporis *in vivo* and tinea unguium (onychomycosis) *ex vivo*.

## Results

### Photodynamic antimicrobial efficacy on microconidia *in vitro*

The cytotoxicity of AE alone and 435 ± 10 nm light irradiation alone on the survival rate of *T. rubrum* microconidia was firstly investigated using MTT assay. Microconidia were incubated with 10 μM of AE in the dark for 1, 2 and 3 h, respectively, and there was no significant difference between the survival rates of microconidia treated with AE alone and that without any treatment (*P* > 0.05; Fig. 1C). The microconidia were irradiated with 2.4, 12, 24, 48 and 72 J cm^−2^ of 435 ± 10 nm light, respectively, and no obvious decrease in the survival rate was observed (*P* > 0.05; Fig. 1D). These results indicated that AE alone and light irradiation alone had no obvious cytotoxicity against *T. rubrum* microconidia.

Next, *T. rubrum* microconidia were incubated with 1 μM AE in the dark for 2 h and irradiated with 435 ± 10 nm light for 0, 1, 5, 10 and 20 min, respectively. As shown in Fig. 1E and F, the survival rates of microconidia decreased with the increasing light energy doses, and 1 μM AE in combination with 2.4, 12, 24, 48 and 72 J cm^−2^ of light irradiation decreased the survival rate of microconidia to 61.86%, 43.98%, 33.46%, 27.08% and 17.10% for control strain, and decrease the survival rate of microconidia to 66.10%, 50.93%, 44.06%, 38.10% and 18.63% for clinical isolate (*P* < 0.01).

Subsequent experiments examined a higher AE concentration (10 μM), and the obtained results were shown in Fig. 1G and H. After treatment with 10 μM AE and irradiated with 2.4, 12, 24 and 48 J cm^−2^, the survival rate of microconidia reduced to 53.03%, 38.17%, 4.75% and 2.83% for *T. rubrum* control strain and reduced to 63.30%, 45.60%, 13.30% and 7.16% for *T. rubrum* clinical strain. When 72 J cm^−2^ of light was applied, the survival rate decreased to 0% for both *T. rubrum* strains, demonstrating that all microconidia were inactivated.

### Inhibitory effect of AE‐mediated aPDT

The inhibitory effect of AE‐mediated aPDT on growth of *T. rubrum* is shown in Fig. 1I and J. Normal colony grown was observed in the absence of AE and irradiation. Following treatment with 0.1 μM of AE and irradiation with 48 J cm^−2^ light, the colony diameters of the two *T. rubrum* strains were slightly lower compared with that without any treatment. Significant decrease in colony diameters of *T. rubrum* was observed after 1 μM AE‐mediated aPDT treatment, demonstrating that it had a strong inhibitory effect on the growth of *T. rubrum*. When the concentration of AE reached to 10 μM, no colony was developed after 7 days of incubation.

### AE intracellular location

**Fig. 2:**
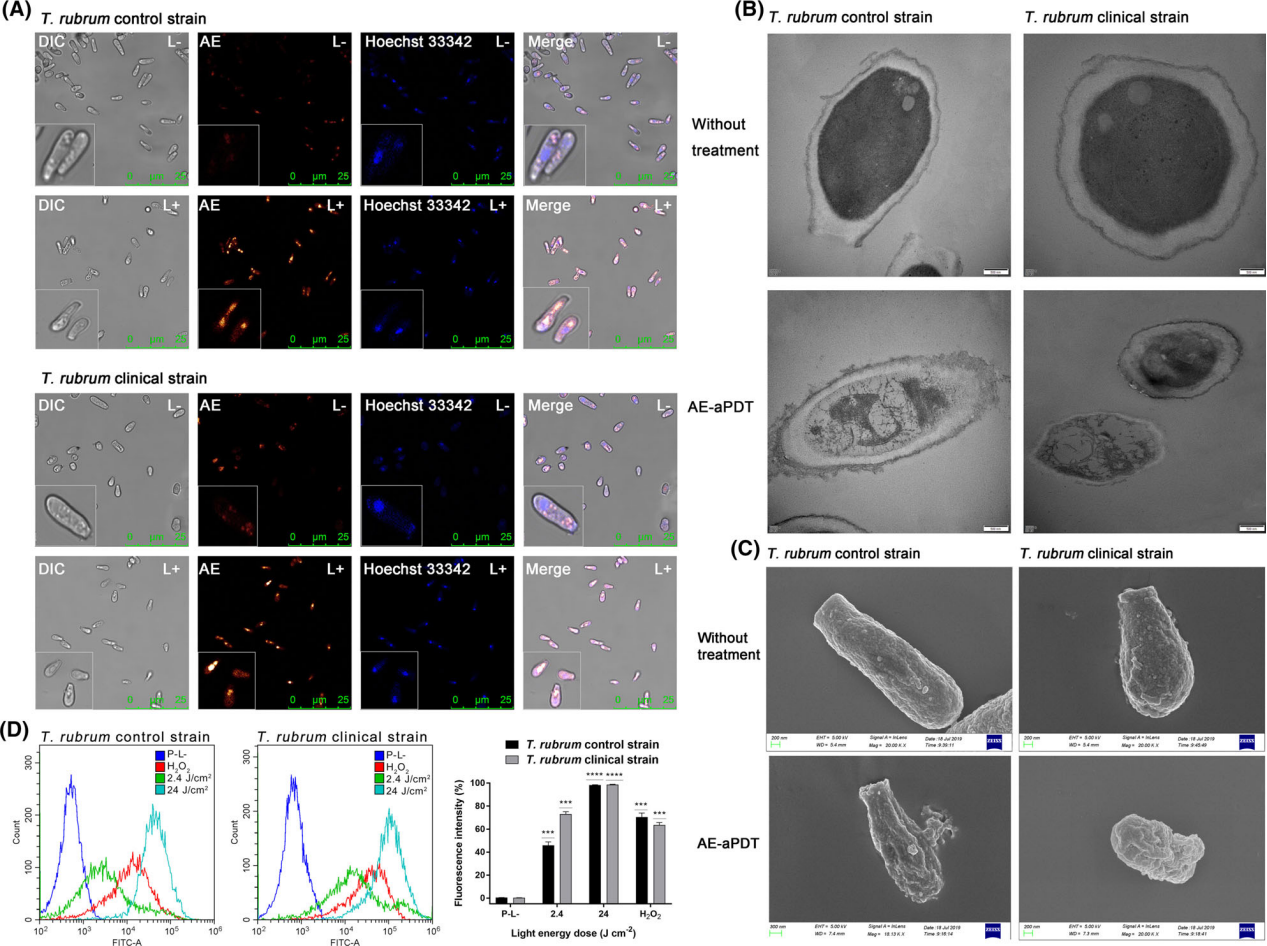
Photodynamic effects of AE on microconidia of *T. rubrum* control and clinical strains. A. Confocal laser scanning microscope images of *T. rubrum* microconidia treated with 10 μM AE in the dark for 2 h (L−) and treated with 10 μM AE in the dark for 2 h and irradiated with 48 J cm^−2^ of 435 ± 10 nm light (L+); the nucleus was stained with 1 μg ml^−1^ of Hoechst 33342 at room temperature for 10 min. B. Representative transmission electron microscope images of *T. rubrum* microconidia without any treatment and treated with AE‐mediated aPDT (10 μM of AE, 48 J cm^−2^ of light irradiation). C. Representative field emission scanning electron microscope images of *T. rubrum* microconidia without any treatment and treated with AE‐mediated aPDT (10 μM of AE, 48 J cm^−2^ of light irradiation). D. AE‐mediated aPDT induced intracellular ROS accumulation in *T. rubrum* microconidia; (left): *T. rubrum* control strain; (middle): *T. rubrum* clinical strain; (right): fluorescence intensities of DCF in *T. rubrum* microconidia (P−L−: negative control, microconidia without any treatment; 2.4 J cm^−2^: microconidia treated with 1 μM AE and irradiated with 2.4 J cm^−2^ light; 24 J cm^−2^: microconidia treated with 1 μM AE and irradiated with 24 J cm^−2^ light; H_2_O_2_: positive control, microconidia treated with 10 mM H_2_O_2_ for 1 h). Each value refers to mean ± standard deviation (SD) (*n* = 3) (ns: no significance, *P < 0.05, **P < 0.01, ***P < 0.001).

Confocal laser scanning microscopy and a DNA‐specific fluorescent dye were used to elucidate the intracellular location of AE in *T. rubrum* microconidia. As shown in Fig. 2A, spots of weak red fluorescence from AE could be observed in the cytoplasm of *T. rubrum* microconidia after incubation in the dark for 2 h (AE, L−), and the nucleus stained with Hoechst 33342 could be distinguished as blue punctate fluorescence (Hoechst 33342, L−). In contrast, following irradiation with 48 J cm^−2^ light, stronger red fluorescence of AE diffused throughout the entire cytoplasm of *T. rubrum* microconidia (AE, L+), but it did not overlap the blue fluorescence from Hoechst 33342 (Merge, L+).

### Effect of AE‐mediated aPDT on cellular structure

High‐resolution transmission electron microscope was employed to evaluate the effects induced by AE‐mediated aPDT on cellular structure of microconidia, and representative images were shown in Fig. 2B. Normal cellular structure was observed in microconidia of two *T. rubrum* strains without any treatment (P−L−). In these images, the cell wall and cytoplasm membrane were intact, the nucleus was well distinguished, and the ribosomes were dispersed in cytoplasm as dark particles. In contrast, after treatment with 10 μM AE in combination with 48 J cm^−2^ light irradiation, obvious damage was observed on the cell wall, and the cell envelop became thinner. Although the cytoplasm membrane was still intact, the intracellular matrix was condensed and nucleus was severely damaged (P + L+).

### Effect of AE‐mediated aPDT on microconidial surface

The effects induced by AE‐mediated aPDT on microconidial surface were investigated by field emission scanning electron microscopy, and representative images were shown in Fig. 2C. The short rod‐shaped *T. rubrum* microconidia with intact surface were observed for two *T. rubrum* strains without any treatment (P−L−). After treatment with 10 μM AE and irradiation with 48 J cm^−2^, the surface of microconidia was still intact but became twisted.

### Generation of intracellular ROS

Flow cytometry and ROS probe H_2_DCFDA were employed to detect the generation of intracellular ROS in *T. rubrum* microconidia induced by AE‐mediated aPDT. H_2_DCFDA is a non‐fluorescent probe which can be hydrolysed by endogenous esterases, and its de‐esterified product can be transferred to a fluorescent 2′,7′‐dichlorofluorescein (DCF) under oxidation of ROS. As shown in Fig. 2D, compared to the microconidia without any treatment (P−L−, negative control), the ROS level in microconidia increased by 45.85% and 73.10% for the control and clinical *T. rubrum* strains after treatment with 1 μM AE and irradiation with 2.4 J cm^−2^ light, respectively. Following 24 J cm^−2^ light irradiation, 1 μM AE could increase the ROS level in microconidia by about 98.50% for the two *T. rubrum* strains. As the positive control, the ROS level in microconidia incubated with 10 mM H_2_O_2_ for 1 h increased by 70.45% and 63.66% for the control and clinical *T. rubrum* strains, respectively.

### AE‐mediated aPDT treatment efficacy against tinea corporis *in vivo*

**Fig. 3:**
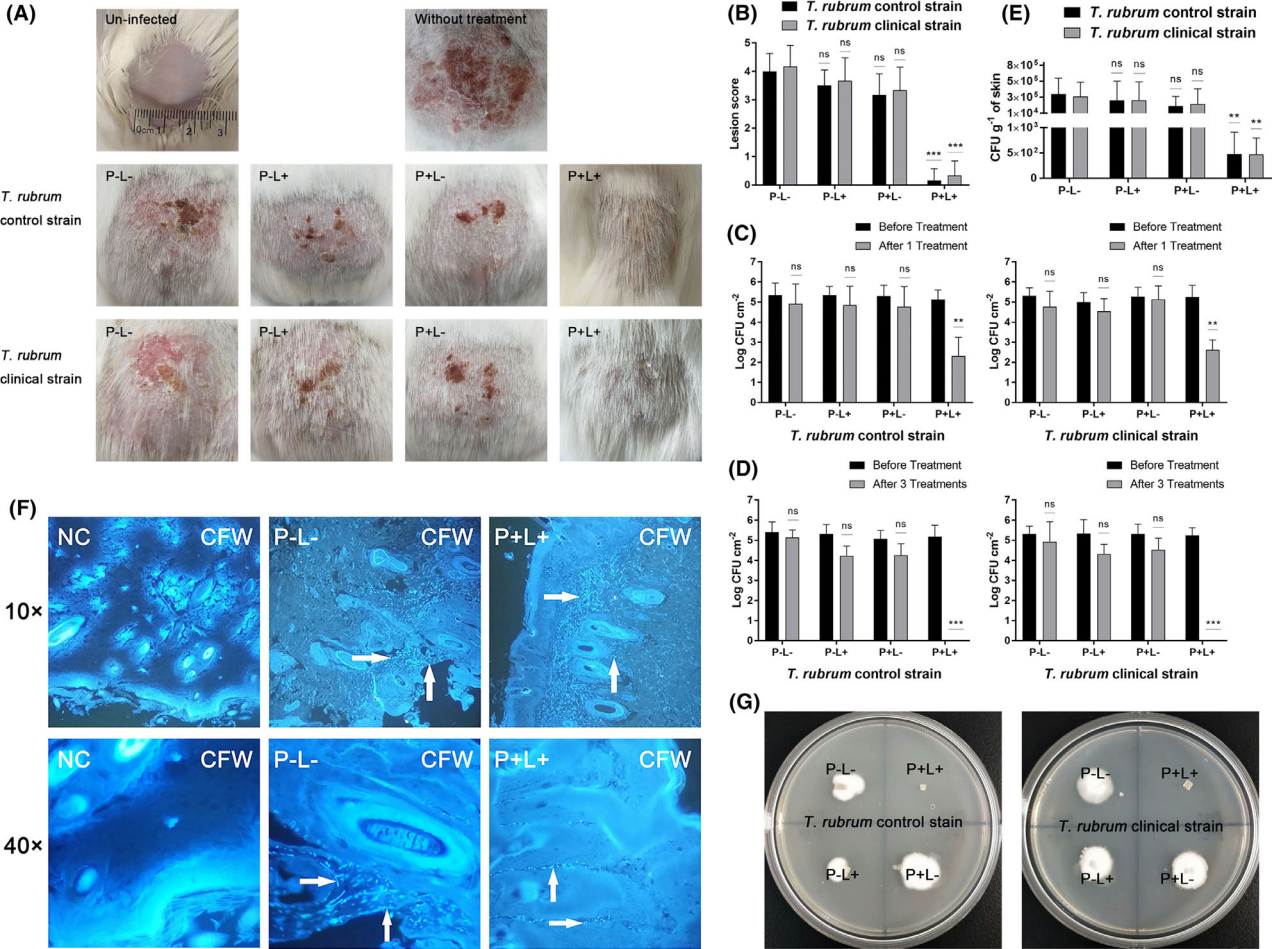
Photodynamic treatment effects of AE for tinea corporis *in vivo* and tinea unguium *ex vivo*. A. Photographs of infected skin on the dorsum of guinea pigs; (P−L−): treated with sterile PBS; (P−L+): irradiated with 48 J cm^−2^ of 435 ± 10 nm light; (P + L−): treated with 10 μM AE in the dark for 2 h; (P + L+): treated with 10 μM AE in the dark for 2 h and irradiated with 48 J cm^−2^ light. B. Lesion score of infected skin on the dorsum of guinea pigs. C. The amounts of *T. rubrum* cells on the surface of infected skin after 1 time treatment. D. The amounts of *T. rubrum* cells on the surface of infected skin after 3 times of treatments. E. The amount of *T. rubrum* cells in the infected skin tissue after 3 times of treatments. F. CFW staining images of un‐infected skin (NC), infected skin treated with sterile PBS (P−L−) and infected skin treated with 10 μM AE and irradiated with 48 J cm^−2^ light for 3 times (P + L+). G. Effectiveness of AE‐mediated aPDT in onychomycosis *ex vivo* model. (P−L−): treated with sterile PBS; (P−L+): irradiated with 48 J cm^−2^ of 435 ± 10 nm light; (P + L−): treated with 10 μM AE in the dark for 2 h; (P + L+): treated with 10 μM AE in the dark for 2 h and irradiated with 48 J cm^−2^ light. Each value refers to mean ± standard deviation (SD) (*n* = 3) (ns: no significance, *P < 0.05, **P < 0.01, ***P < 0.001).

To assess the AE‐mediated aPDT treatment efficacy *in vivo*, a tinea corporis guinea pig model was established referring to a previously reported study (Garvey *et al*., 2015). The photographs of infected skin for the 4 experimental groups were taken on the 10th day (Fig. 3A). From these photographs, it was shown that the skin lesions in light‐irradiated group (P−L+) and AE‐treated group (P + L−) were similar to that in group without any treatment (P−L−). Partial skin damage, severe scab and hair loss could be clearly observed on the infected skin in these groups. After AE‐mediated aPDT treatment (P + L+), skin damage and scab could not be found, and the growth of hair could be observed. The lesion scores of each group were calculated and shown in Fig. 3B. There was no significant difference in the lesion score among the P−L+, P + L− and P−L− groups. In contrast, the lesion score in the P + L+ group was significantly lower compared with that in other groups.

The amounts of *T. rubrum* cells on the infected skin surface of guinea pigs were further investigated. As shown in Fig. 3C, there were over 5 log_10_ *T. rubrum* cells per cm^2^ on the infected skin surface before treatments. After treatment with 10 μM AE alone or irradiated with 48 J cm^−2^ alone, the fungal cells did not significantly decrease (*P* > 0.05). After treatment with 10 μM AE in combination with 48 J cm^−2^ light for one time, the fungal cells decreased over 2 log_10_ per cm^2^ skin (*P* < 0.01). Following treatment with AE‐mediated aPDT three times, no viable *T. rubrum* cells were detected on the infected skin surface (*P* < 0.001, Fig. 3D).

We further investigated the amount of *T. rubrum* cells in the infected skin tissue, and the obtained results were shown in Fig. 3E. The fungal cells in the infected skin tissue of the P−L−, P−L+ and P + L− groups were approximately 3 × 10^5^ CFU *T. rubrum* cells g^−1^. After treatment with 10 μM AE and irradiation with 48 J cm^−2^ light three times, the fungal cells in the infected skin tissue decreased to 5 × 10^2^ CFU *T. rubrum* cells g^−1^ (*P* < 0.01).

The infected skin tissue was sectioned, stained with CFW and observed on a fluorescent microscope. As shown in Fig. 3F, no microconidia and hypha of *T. rubrum* were found in the skin tissue of un‐infected guinea pig (NC). In the *T. rubrum*‐infected guinea pig without any treatment (P−L−), microconidia and hypha could be detected not only on the skin surface but also in the skin tissue. After treatment with 10 μM AE and irradiation with 48 J cm^−2^ light three times, *T. rubrum* microconidia did not exist on the skin surface, but there was still few microconidia present in the deep skin tissue.

### AE‐mediated aPDT treatment effect against onychomycosis *ex vivo*

Tinea unguium (onychomycosis) *ex vivo* model was established using *T. rubrum* to evaluate AE‐mediated aPDT treatment effect. As shown in Fig. 3G, the growth of *T. rubrum* colony could be observed for the infected nail piece without any treatment (P−L−), irradiated with 48 J cm^−2^ light alone (P−L+) and treated with 10 μM AE alone (P + L−) after 28 days of incubation at 28°C. However, after treated with 10 μM AE and irradiated with 48 J cm^−2^ light, the growth of *T. rubrum* colony was not found for the aPDT‐treated nail piece after 28 days of incubation.

## Discussion

Considerable attention has been initially focused on the application of aPDT to treat antibiotic‐resistant bacterial infections because its unique inactivation mode is unlikely to induce any resistant mechanism. However, on account of the limited treatment depth, aPDT is more suitable for the treatment of superficial infections, such as dermatophytosis caused by *T. rubrum*. Previous studies have demonstrated that aPDT mediated by several classical PSs, including MB (Rodrigues *et al*., 2012; Li *et al*., 2021), toluidine blue (TBO) (Rodrigues *et al*., 2012; Baltazar Lde *et al*., 2013), RB (Houang *et al*., 2018, 2019), new methylene blue (NMB), indocyanine green (ICG) (Fekrazad *et al*., 2017), porphyrin, Eosin Y (Shamali *et al*., 2018), phthalocyanine (Lam *et al*., 2014) and curcumin (Brasch *et al*., 2018, 2017), could effectively inactivate microconidia or hypha of *T. rubrum in vitr*o. The susceptibility of *T. rubrum* strains to aPDT was not impaired or affected in any way by their resistance to antifungal agents (Shen *et al*., 2021). Additionally, the combination of aPDT and low dose antifungals had an enhanced synergetic antimicrobial effect against *T. rubrum*, which had the potential to reduce the treatment times, drug dosages, drug toxicity and improve patient compliance (Morton *et al*., 2014). All of these studies pointed out that aPDT could be used as an alternative therapeutic option or in synergy with current available antifungals for the treatment of dermatophytosis caused by *T. rubrum*.

AE is a natural anthraquinone PS isolated from traditional Chinese herbs. Although we have previously found that AE in the presence of light could effectively inactivate drug‐resistant *Candida albicans* and *Acinetobacter baumannii* (Li *et al*., 2020; Ma *et al*., 2020), whether the photodynamic antimicrobial efficacy of AE against dermatophytes and it can be used in the photodynamic treatment of dermatophytosis remain unclear. The results obtained in the present study showed that under irradiation with 72 J cm^−2^ blue light, 1 μM AE could decrease the survival rate of microconidia by over 80%, and 10 μM AE could inactivate all microconidia, indicating that AE was a potent PS against *T. rubrum*. Paz‐Cristobal *et al*. reported that 10 μM hypericin irradiated with 37 J cm^−2^ light yielded about 2.5 log_10_ decrease in survival of *T. rubrum* microconidia, which was determined by colony‐forming unit (CFU) assay (Paz‐Cristobal *et al*., 2014). Although AE is quite similar to hypericin in chemical structure, and the control *T. rubrum* strain (ATCC 28188) was employed in both studies, the photodynamic antimicrobial efficacy and merits of AE and hypericin were hardly comparable because of the differences in cell densities of microconidia and determination methods of the two studies. Li *et al*. used both CFU and MTT assays to evaluate the antifungal effect of MB‐mediated aPDT against *T. rubrum* and found that MTT assay was consistent with CFU assay, but more convenient and time‐saving (Li *et al*., 2021).

We also found that AE‐mediated aPDT demonstrated a growth‐inhibiting effect on both *T. rubrum* strains, and remarkable reduced diameters of colonies were observed, which was consistent with the previously reported study (Kamp *et al*., 2005). The confocal laser scanning microscope images demonstrated that spots of weak red fluorescence from AE were located in the cytoplasm but were not oriented to in the cell envelop of microconidia before light irradiation, and stronger AE fluorescence was detected throughout the entire cytoplasm of microconidia after irradiation, suggesting that AE initially targeted to the organelles in cytoplasm and caused damage nearby, which lead to the diffusion of AE fluorescence. However, which organelle is the target of AE still needs further investigation.

Our team previously found that haematoporphyrin monomethyl ether (HMME), a second‐generation porphyrin‐based PS developed in China, initially bound to the cell wall and membrane of *T. rubrum* microconidia, forming a periphery fluorescence pattern, but it could not enter into the cytoplasm of microconidia. After irradiation, fluorescence from HMME could be found in the entire cell, suggesting that the cell envelop of *T. rubrum* microconidia might be target of HMME and it was disturbed during HMME‐mediated aPDT treatment (Pan *et al*., 2020). In several previous studies, Morton *et al*. (2014) found that RB targeted to the cell membrane of *T. rubrum* microconidia. However, Lam *et al*. (2014) reported that Pc 4 red fluorescence primarily was located on the cell wall and in cytoplasm organelles (probably mitochondria) of microconidia. Paz‐Cristobal *et al*. (2014) discovered that hypericin was mainly diffused in the cytoplasm but not nucleus of *T. rubrum* microconidia, which was quite similar to the obtained results of this study. The different targets of these PSs might be due to their differences in polarity and chemical structure (Morton *et al*., 2014).

Details of damage induced by AE‐mediated aPDT on cellular structure of *T. rubrum* microconidia were provided by transmission electron microscope images. From these images, we found that AE‐mediated aPDT mainly caused damage to the cytoplasm of microconidia. Although the cytoplasmic membrane and cell wall became thinner, they were still intact after light irradiation, which was in accordance with the results obtained by confocal laser scanning microscopy. Field emission scanning electron microscopy showed that the microconidial surface was intact but became twisted after AE‐mediated aPDT treatment, suggesting that AE‐mediated aPDT did not induce severe damage to the cell envelop. Similar results were also reported by Smijs *et al*. (2008), who found that Sylsens B‐mediated aPDT could cause reversible cell wall deformations and bulge formation of *T. rubrum* microconidia.

H_2_DCFDA was employed as a ROS probe combined with flow cytometry to evaluate the level of intracellular ROS in microconidia induced by AE‐mediated aPDT. The results exhibited that treatment with 1 μM AE and irradiation with 24 J cm^−2^ light could yield 98.5% increase in intracellular ROS level. However, incubation with 10 mM H_2_O_2_ for 1 h only caused about 70% increase in intracellular ROS level, indicating that AE‐mediated aPDT increased levels of intracellular ROS. These ROS species reacted with various intracellular components, resulting in the peroxidation of lipids and disruption of structural proteins and enzymes, subsequently leading to the death of fungal cells (Baltazar Lde *et al*., 2013).

Although a number of studies reported that microconidia or hypha of *T. rubrum* were sensitive to the lethal aPDT mediated by various PSs *in vitro*, aPDT treatment efficacy against *T. rubrum*‐caused dermatophytosis has been rarely investigated *in vivo*. Smijs *et al*. (2007, 2008) previously developed an *T. rurbum*‐infected human stratum corneum (SC) *ex vivo* model by inoculating its microconidia on the SC of human skin and assessed the treatment efficacy of Sylsens B‐mediated aPDT. Baltazar *et al*. (2015) created a *T. rubrum*‐infected C57BL/6 mice model of dermatophytosis and found that TBO‐mediated aPDT could effectively reduce the signs of dermatitis and fungal burden, as well as recover skin tissue architecture. In this work, the *T. rubrum*‐caused tinea corporis guinea pig model was established according to a previously reported study (Garvey *et al*., 2015) and used to assess the treatment efficacy of AE‐mediated aPDT. The obtained results demonstrated that AE‐mediated aPDT is effective against *T. rubrum*‐induced tinea corporis. Skin lesion of AE‐mediated aPDT‐treated guinea pigs was greatly improved compared with that of untreated, AE‐treated and light‐irradiated guinea pigs. The lesion score of aPDT‐treated guinea pigs was also much lower in comparison with that of other animals. We found that three treatments of AE‐mediated aPDT could inactivate all fungal cells on the infected skin surface of guinea pigs. However, using tissue homogenate and CFW stain, we found that a small number *T. rubrum* microconidia were existed in the skin tissue, which might cause the recurrence of tinea corporis. We speculated that this result might be due to the low permeability of AE in skin tissue.

Previously, *ex vivo* model has been frequently used to evaluate the treatment efficacy of aPDT against onychomycosis (Smijs and Pavel, 2011). Hollander *et al*. (2015) inoculated microconidia of clinically isolated *T. rubrum* in healthy human nails to induce onychomycosis *ex vivo* model and discovered that multifunctional porphyrin‐mediated aPDT could effectively cure onychomycosis. Mehra *et al*. (2015) demonstrated that TBO‐mediated aPDT effectively inhibited the growth of *T. rubrum* in onychomycosis *ex vivo* model. In line with these previous studies, the present study used healthy human nail pieces and microconidia of *T. rubrum* to establish tinea unguium *ex vivo* model and investigated the photodynamic treatment effect of AE for onychomycosis. Although the results obtained demonstrated that AE‐mediated aPDT had an effective therapeutic effect for onychomycosis, this *ex vivo* model still could not simulate the real infection of *T. rubrum* in nails. In 2011, Shimamura *et al*. (2011) treated rabbit with methylprednisolone for four weeks, dripped microconidial suspension of *Trichophyton mentagrophytes* onto the nail at a site between the lunula and the proximal nail fold and successfully established a rabbit onychomycosis *in vivo* model to evaluate the treatment efficacy of ciclopirox and amorolfine. Recently, they have developed a guinea pig onychomycosis *in vivo* model using the similar method (Hasegawa and Shibuya, 2020). In reference to these studies, we have recently developed a *T. rubrum*‐induced rabbit onychomycosis *in vivo* model, which will be used to assess the treatment efficacy of AE‐mediated aPDT against onychomycosis in our ongoing studies.

Even if almost all *in vitro*, *ex vivo* and *in vivo* studies have shown that aPDT is a promising alternative for the treatment of dermatophytosis caused by *T. rubrum*, its clinical application is still facing obstacles. The most critical one is that PS cannot permeate skin tissue and nails adequately, resulting in the reduction in photodynamic activity and recurrence of dermatophytosis (Bhatta *et al*., 2016). In case of onychomycosis, satisfied therapeutic outcomes were obtained only after pretreatment of nails with microabrasion or 20–40% urea (Simmons *et al*., 2015). Therefore, research attention has been focused on how to improve the permeability of PS in the skin tissue and nails. It has been reported that solid‐lipid nanoparticles, liposome, transfersome, niosome, nanoemulsion, nanogel, micelle, *etc*., can be used as topical drug delivery system of antifungals for the treatment of superficial fungal infections (Garg *et al*., 2020). Transfersome is a highly deformable nano‐carrier possessing ability to transport drug molecules through the narrow pathways of skin cell membrane (5–10 times narrower than cell diameter) without significant loss, driven by the transcutaneous water gradient (Vikas *et al*., 2020). Sigurgeirsson *et al*. indicated that terbinafine in transfersome (TDT 067) could accumulate higher concentration of terbinafine in the nail plate and achieve higher therapeutic efficacy against onychomycosis than conventional terbinafine (Sigurgeirsson and Ghannoum, 2012), suggesting that transfersome can be employed as a promising delivery system of AE to enhance its permeability in the skin tissue and nails, making AE‐mediated aPDT more efficient, convenient and time‐saving. Biosafety assessments are needed to verify that AE can satisfy the requirements of clinical practice. Novel AE encapsulated transfersome formulations will be developed, and the cytotoxicity and biosafety of AE will be investigated using relevant mammalian skin cell lines and animal models in our future research.

In conclusion, the present study investigated the efficacy and effects of aPDT mediated by AE on *T. rubrum* microconidia *in vitro* and evaluated the treatment efficacy of AE‐mediated aPDT for *T. rubrum*‐caused tinea corporis *in vivo* and tinea unguium *ex vivo*. AE exhibited no significant dark toxicity against microconidia of *T. rubrum* at the used concentration, but in the presence of light, it effectively inactivated *T. rubrum* microconidia in a light energy dose‐dependent manner and exhibited a strong inhibiting effect on the growth of *T. rubrum*. Confocal laser scanning microscope, transmission electron microscope and field emission scanning electron microscope images indicated that AE mainly targeted to the organelles in cytoplasm but not cell envelop of microconidia, and caused severe damage to the cytoplasm of microconidia after light irradiation through the generation of abundant intracellular ROS, which was confirmed by flow cytometry. Additionally, AE‐mediated aPDT possessed an effective therapeutic effect for the *T. rubrum*‐caused tinea corporis on guinea pig model. After 3 AE‐mediated aPDT treatments, the skin lesion was greatly improved and *T. rubrum* cells on the infected skin surface were all killed, but a few microconidia still existed in the deep skin tissue, which might be due to the low permeability of AE in skin tissue. AE‐mediated aPDT also had an effective treatment effect for onychomycosis in *ex vivo* model. These results obtained suggest that AE is a promising PS for the photodynamic treatment of dermatophytosis caused by *T. rubrum*. And we will develop novel AE encapsulated transfersome formulations to further enhance its permeability in the skin tissue and nails, and assess the cytotoxicity and biosafety of AE i*n vitro* and *in vivo* in our subsequent studies.

## Experimental procedures

### *T*. *rubrum* strains and preparation of microconidia

Two *T. rubrum* strains were used in this study: a control strain (ATCC 28188) was obtained from the Shaanxi Provincial Center for Disease Control and Prevention, Xi’an, China, and a clinical isolate was gathered from the infected toenail from a patient with onychomycosis in the Department of Dermatology, the Second Affiliated Hospital of Xi’an Jiao Tong University, Xi’an, China.

Microconidia of two *T. rubrum* strains were prepared according to a previously reported study (Lam *et al*., 2014). Briefly, the two strains were inoculated on potato dextrose agar (PDA, Solarbio, Beijing, China) supplemented with 0.025% Sabouraud dextrose broth (SDB, Solarbio) and 1.0% penicillin–streptomycin (HyClone, Logan, UT, USA). After cultured at 28°C for at least 10 days to sufficiently produce microconidia, a sterile cotton tip applicator was used to transfer the microconidia from the surface of white cotton‐like growth (hyphae) colonies into sterile phosphate‐buffered saline (PBS). The microconidia were separated by filtration of the suspension through sterile cotton gauze and diluted to a density of 1 × 10^8^ conidia ml^−1^ in PBS with a haemocytometer.

### Photosensitizer

AE with purity of 99% was provided by the Nanjing Jingzhu Biotechnology, China. To prepare 10 mM stock solution, 2.70 mg of AE was dissolved in 50 μl of dimethyl sulfoxide (DMSO) and diluted to 1 ml using sterile PBS. The prepared solution heated to 45°C with continuously stirring until it was completely dissolved as indicated by total transparency of the solution. Next, the AE stock solution was diluted to the desired concentrations using sterile PBS for subsequent experiments.

### Light instruments

The light illumination experiments were performed on a 50 W xenon lamp (Ceaulight CEL‐HXF‐300, China) equipped with an optical filter for selecting blue light with the wavelength of 435 ± 10 nm. There was a 10‐cm distance between the light source and biological samples. And a power meter (Ceaulight NP‐2000, Beijing, China) was employed to adjust the light fluence rate to 40 mW cm^−2^ at the level of samples.

### Photodynamic inactivation of microconidia *in vitro*

The microconidial suspension of two *T. rubrum* strains (1 ml, 1 × 10^8^ conidia ml^−1^) was centrifuged (3500 rpm, 10 min), and the supernatant was discarded. The remaining microconidial pellets were divided into four groups: microconidia in the first group were resuspended in 10 μM AE in sterile PBS and incubated in the dark for 1, 2 and 3 h in a shaking incubator (150 rpm); the second group microconidia were illuminated directly with 435 ± 10 nm light for 1, 5, 10, 20 and 30 min, corresponding to the light energy doses of 2.4, 12, 24, 48 and 72 J cm^−2^, respectively; microconidia in the third group were treated with 1 μM of AE in the dark for 2 h, and subsequently illuminated with 435 ± 10 nm light for 0, 1, 5, 10 and 20 min, respectively. Microconidia in the last group were treated with 10 μM AE and irradiated with light for 0, 1, 5, 10 and 20 min, respectively. The microconidia of four groups were collected by centrifugation, washed with sterile PBS and resuspended in 1 ml of sterile PBS.

After 4 h following light irradiation, the metabolic activities of microconidia were assessed using tetrazolium salt thiazolyl blue (MTT) assay (Li *et al*., 2021). Briefly, the treated microconidial (200 μl) suspension was transferred into a 96‐well microplate (Corning, USA), and 20 μl of MTT solution was added to each well. After incubation at 37°C for 4 h, the 96‐well plate was centrifuged at 3500 rpm for 10 min, and the supernatant was removed, followed by adding 200 μl of DMSO. The microplate was gently shaken at room temperature for 15 min, and the absorbance at 492 nm was measured by a microplate reader (Synergy HT Biotech, USA). All experiments were performed three times, and the survival rate of *T. rubrum* microconidia was calculated according to Equation 1.

(1) $$\begin{array}{ll} & \text{Survival}\;\text{rate}\;\left(\%\right)=\\ & \frac{{\text{OD}}_{492\;\text{nm}}\;\text{value}\;\left(\text{treated}\;\text{groups}\right)‐{\text{OD}}_{492\;\text{nm}}\;\text{value}\;\left(\text{blank}\right)}{{\text{OD}}_{492\;\text{nm}}\;\text{value}\;\left(\text{control}\;\text{groups}\right)‐{\text{OD}}_{492\;\text{nm}}\;\text{value}\;\left(\text{blank}\right)}\times 100\% \end{array}$$

### *T*. *rubrum* growth inhibition assays

Microconidia of two *T. rubrum* strains were collected by centrifugation at 3500 rpm for 10 min, resuspended in 0, 0.1, 1 and 10 μM of sterile AE solution in the dark for 2 h and illuminated with 435 ± 10 nm light for 20 min (48 J cm^−2^). When the irradiation process was finished, the microconidia were collected by centrifugation, resuspended in sterile PBS and 10‐fold diluted serially. Subsequently, 10 μl of each dilution was evenly spread on three PDA plates and incubated at 28°C for 1 to 7 days. During the incubation process, the diameter of developed colonies was measured by a Vernier calliper each day. The experiment was repeated three times.

### Confocal laser scanning microscopy

Microconidia of two *T. rubrum* strains were collected by centrifugation and incubated with 10 μM of AE in the dark for 2 h. Next, the microconidia were divided into two groups. The first group microconidia were incubated in the dark for additional 20 min, and the second group were illuminated with 435 ± 10 nm light for 20 min (48 J cm^−2^). Following irradiation, microconidia in two group were centrifuged and resuspended in PBS containing 1 μg ml^−1^ of Hoechst 33342 (Sigma‐Aldrich, Shanghai, China). After incubation at room temperature for 10 min and washing with PBS for three times, 10 μl of microconidial samples was spotted onto glass slides, covered with coverslips and observed on a confocal laser scanning microscope (Leica SP8 STED, Leica, Wetzlar, Germany).

### Transmission electron microscopy

Microconidia of two *T. rubrum* strains were treated with 10 μM AE in the dark for 2 h and illuminated with 435 ± 10 nm light for 20 min (48 J cm^−2^). After centrifugation at 3500 rpm for 10 min and removal of the supernatant, the microconidial pellets were immersed in 2.5% glutaraldehyde and fixed at 4°C for 3 h. Following two washes with PBS, the microconidia were treated with 1% osmium tetroxide (Johnson Matthey, London, UK) at 4°C for another 3 h. Then, the microconidia were washed with PBS, immersed in 30–100% ethanol and epoxypropane for dehydration and embedded in Epon 812 epoxy resin (SPI‐Chem, West Chester, PA, USA) at 60°C for 24 h. Next, an LKB‐V ultratome (LKB, Sweden) was used to prepare 50–70 nm thin‐section samples, which were stained with uranyl acetate and lead citrate for 15 min, respectively. The prepared samples were finally viewed on a high‐resolution transmission electron microscope (Hitachi H‐7650, Japan). The microconidia without any treatment were used as control.

### Field emission scanning electron microscopy

Microconidia were treated with AE‐mediated aPDT as described above. The pellets were centrifuged, washed with PBS and transferred into a 24‐well microplate (Corning, USA) containing round sterile coverslips. After incubation at 28°C for 2 h, the coverslips were carefully washed and immersed in 2.5% glutaraldehyde and kept at 4°C for 3 h. Following gentle wash with PBS, the microconidia were treated with 1% osmium tetroxide at 4°C for 3 h. Next, the samples on coverslips were dehydrated with 10–100% ethanol, freeze‐dried and sputter‐coated with gold. The prepared samples were finally viewed on a field emission scanning electron microscope (GeminiSEM 500; ZEISS, Thuringen, Germany). The microconidia without any treatment were used as control.

### AE‐mediated aPDT induced intracellular ROS generation

The intracellular ROS induced by AE‐mediated aPDT was monitored using a ROS probe 2′,7′‐dichlorohydrofluorescein diacetate (H_2_DCFDA, Invitrogen, USA) in combination with flow cytometry. H_2_DCFDA probe (2 mg) was dissolved in 800 μl of DMSO to prepare stock solution. Microconidia of two *T. rubrum* strains were incubated with 1 μM of AE in the dark for 2 h and irradiated with 435 ± 10 nm light for 1 and 10 min (2.4 and 24 J cm^−2^), respectively. After irradiation, the microconidia were washed with PBS, and 10 μl H_2_DCFDA stock solution was added. After incubation at 37°C in the dark for 30 min and washed with PBS, a flow cytometry (Beckman Counter CytoFLEX, Pasadena, China) was employed to measure the intracellular ROS. Microconidia without any treatment were used as negative control, and microconidia incubated with 10 mM H_2_O_2_ for 1 h were used as positive control.

### Establishment of tinea corporis animal model

Tinea corporis animal model was established as a previously reported study with little modification (Garvey *et al*., 2015). In brief, Hartley male guinea pigs (200–250 g) were purchased from the Laboratory Animal Center of the Xi’an Jiao Tong University Health Science Center and maintained in SPF environment at 25°C with free access to food and water. All animal experiments were approved by the Institutional Animal Care and Use Committee of Xi’an Jiao Tong University. The guinea pigs were daily immune‐suppressed by intraperitoneal injection of cyclophosphamide (0.1 g kg^−1^) for 3 days. Next, the animals were anaesthetized with 10% (w/v) chloral hydrate solution (1 ml kg^−1^), and the hair on dorsum was scraped with electric scissors and depilatory cream. An abrasion area with 2.0 × 2.0 cm on the dorsum of guinea pigs was made by pre‐autoclaved abrasive paper, and 100 μl of *T. rubrum* microconidial suspension (1 × 10^7^ conidia ml^−1^) was inoculated on the abrasion area. After inoculation, each animal was continuously maintained in SPF environment for 3 days and injected with cyclophosphamide daily.

### AE‐mediated aPDT treatment of tinea corporis *in vivo*

After successful infection by *T. rubrum*, 24 guinea pigs were randomly divided into four groups: guinea pigs in the first group were treated with 100 μl of sterile PBS (P−L−, *n* = 6); guinea pigs in the second group were treated with 100 μl of AE PBS solution (10 μM) in the dark for 2 h (P + L−, *n* = 6); guinea pigs in the third group were irradiated with 48 J cm^−2^ of 435 ± 10 nm light (P−L+, *n* = 6); guinea pigs in the last group was treated with 100 μl of AE in the dark for 2 h and irradiated with 48 J cm^−2^ light (P + L+, *n* = 6). The treatments as described above were started 3 days after *T. rubrum* infections, once a day for three consecutive days, and the therapeutic effect was assessed on the 10th day.

### Clinical evaluation

The treatment effects of AE‐mediated aPDT for tinea corporis were clinically evaluated according to a previously reported study (Mei *et al*., 2015) and recorded using a lesion score as follows: 0 = no skin lesions, hair growth; 1 = a small amount of mild erythema on the skin; 2 = marked redness and swelling; 3 = obvious redness, scabby and sparse hair; 4 = partial skin damage, severe scab, hair loss; and 5 = large area of skin damage and hair loss. The scores of guinea pigs in each group were counted to determine the treatment efficacy of AE‐mediated aPDT, which was calculated as Equation 2.

(2) $$\text{Lesion}\;\text{score}=\frac{\sum (n=6)}{6}$$

### Determination of fungal cells on/in the skin

To determine the amount of *T. rubrum* cells on the skin surface of guinea pigs, a sterile PBS‐moistened cotton swab was used to sufficiently smear the infected area and placed into 1 ml sterile PBS in Falcon tube and vortexed for 10 min. The *T. rubrum* suspension was serially 10‐fold diluted, and 10 μl of each dilution was equably spread on three PDA plates supplemented with 0.025% SDB and 1.0% penicillin–streptomycin. Following incubation at 28°C for 10 days, the developed colonies were counted to calculate the amount of *T. rubrum* cells on the skin surface. Next, the guinea pigs were anaesthetized and euthanized, and the infected skin tissue was excised and divided into two groups. The first group of skin tissue was weighted and homogenized in 1 ml of sterile PBS using a tissue grinder. The tissue homogenate was serially 10‐fold diluted, and 10 μl of each dilution was equably spread on three PDA plates containing SDB and penicillin–streptomycin. Following incubation at 28°C for 10 days, the developed colonies were counted to calculate the amount of *T. rubrum* cells in the skin tissue. The second group skin tissue was fixed in 10% formalin for 24 h, embedded in paraffin, sectioned and stained with Calcofluor white (CFW). The prepared samples were finally observed and recorded on a fluorescent microscope (Carl Zeiss Axio Scope. A1, China).

### Preparation of onychomycosis *ex vivo* model

The tinea unguium (onychomycosis) *ex vivo* model was prepared according to a previous study (Mehra *et al*., 2015). In brief, healthy human nails were provided by volunteers from an anonymous source. The nails were washed with 70% ethyl alcohol in an ultrasonic water bath for 3 min and stored in sealed plastic bags at room temperature no longer than 2 months. The cleaned nails were cut into 7–10 mg rectangular pieces and washed with 70% isopropyl alcohol and autoclaved H_2_O for three times, respectively. Next, each human nail pieces were incubated with 100 μl of *T. rubrum* microconidial suspension (1 × 10^7^ conidia ml^−1^) for 3 h and transferred to the wells of a 24‐well microplate and incubated at 28°C in a humid condition for 7–21 days. To confirm the fungal growth, nail pieces were plated onto PDA plates containing SDB and penicillin–streptomycin at 3, 7, 14 and 21 days after induction of infection.

### AE‐mediated aPDT treatment of onychomycosis *ex vivo*

For aPDT treatment, 6 nail pieces at 21 days after infection were treated with 10 μM of AE in the dark for 2 h and irradiated with 435 ± 10 nm light for 20 min (48 J cm^−2^). Following treatment, the nail pieces were plated onto PDA plates containing SDB and penicillin–streptomycin and incubated at 28°C for additional 30 days. The nail pieces without any treatment (P−L−, *n* = 6) were used as negative control, and the nail pieces treated with 10 μM AE alone (P + L−, *n* = 6) or irradiated with 48 J cm^−2^ light alone (P−L+) were used as positive control.

### Statistical analysis

At least three independent experiments were performed where stated. The data obtained were analysed using GraphPad Prism software and shown as the mean ± standard error of the mean (SEM). Data were compared using one‐way or two‐way ANOVA, and the difference was considered significant in the case of *P* < 0.05.

## Conflict of interest

None declared.
